# Dietary Supplementation with Sea Buckthorn Berry Puree Alters Plasma Metabolomic Profile and Gut Microbiota Composition in Hypercholesterolemia Population

**DOI:** 10.3390/foods11162481

**Published:** 2022-08-17

**Authors:** Kang Chen, Fangfei Zhou, Jian Zhang, Pin Li, Yumei Zhang, Baoru Yang

**Affiliations:** 1 Food Sciences, Department of Life Technologies, University of Turku, 20014 Turku, Finland; 2 Department of Nutrition and Food Hygiene, School of Public Health, Beijing University, Beijing 100191, China; 3 Beijing Key Laboratory of Toxicological Research and Risk Assessment for Food Safety, Beijing 100191, China; 4 Shanxi Center for Testing of Functional Agro-Products, Shanxi Agricultural University, Taiyuan 030031, China

**Keywords:** hypercholesterolemia population, *Hippophaë rhamnoides*, sea buckthorn berries, plasma metabolomic profile, gut microbiota

## Abstract

Sea buckthorn berries have been reported to have beneficial effects on plasma lipid profile and cardiovascular health. This study aimed to investigate the impact of intervention with sea buckthorn berry puree on plasma metabolomics profile and gut microbiota in hypercholesterolemic subjects. A total of 56 subjects with hypercholesterolemia consumed 90 g of sea buckthorn berry puree daily for 90 days, and plasma metabolomic profile was studied at 0 (baseline), 45, and 90 days of intervention by using proton nuclear magnetic resonance spectroscopy (^1^H NMR). Gut microbiota composition was analyzed at the baseline and after 90 days of supplementation by using high-throughput sequencing. The plasma metabolic profile was significantly altered after 45 days of intervention as compared to the baseline (day 0). A clear trend of returning to the baseline metabolomic profile was observed in plasma when the intervention extended from 45 days to 90 days. Despite this, the levels of several key plasma metabolites such as glucose, lactate, and creatine were lowered at day 90 compared to the baseline levels, suggesting an improved energy metabolism in those patients. In addition, intervention with sea buckthorn puree enriched butyrate-producing bacteria and other gut microbes linked to lipid metabolisms such as *Prevotella* and *Faecalibacterium* while depleting *Parasutterella* associated with increased risks of cardiovascular disease. These findings indicate that sea buckthorn berries have potential in modulating energy metabolism and the gut microbiota composition in hypercholesterolemic patients.

## 1. Introduction

Hypercholesterolemia is one of the most important risk factors for cardiovascular disease (CVD) which is the leading cause of death in global diseases [1]. Diet and genetic background as well as diseases such as obesity and type 2 diabetes contribute to elevated levels of cholesterols in the blood [2]. Healthy food choices have been considered an important approach to reducing the risk of CVD. Natural products and phytochemicals with beneficial effects on CVD, such as phenolic compounds from fruits and vegetables, have increasingly drawn attention. Epidemiological and interventional studies have indicated that vegetables and fruits, especially berry meals, have the potential to promote cardiovascular health [3,4,5].

Sea buckthorn (*Hippophaë rhamnoides*) berries are rich in bioactive compounds such as flavonoids, vitamins, phenolic acids, and carotenoids [6]. Feeding sea buckthorn berries or polyphenols extract have shown the beneficial modulatoy effects on carbohydrate and lipid metabolisms. Feeding daily 7–28 mg/kg body weight polyphenols extract from sea buckthorn berries to rats with hyperlipidemia for 5 weeks has reduced serum lipids and inflammatory markers as well as alleviated vascular impairment by decreasing the expression of eNOS, ICAM-1, and LOX-1 in aortas [7]. Feeding 25–100 mg/kg body weight sea buckthorn berries per day for 10 days decreased blood glucose level and insulin resistance in db/db mice by improving pancreas and revering of insulin resistance and quebrachitol present in sea buckthorn might have contributed to the effects [8]. Intake of sea buckthorn freeze-dried powder (400 mg/kg body weight per day) for 10 weeks improved lipid metabolism in obese mice via downregulating the expression of lipogenic genes including SREBP-1c, PPAR-γ, ACC, and SCD1, and upregulating gene expression of enzymes of fatty acid β-oxidation pathways including HSL, CPT-1, and ACOX [9]. A review has summarised that the mechanisms of the effect of sea buckthorn berries on cardiovascular health can be classified as regulating lipid metabolism and decreasing platelet aggregation and inflammation [10]. However, there were contradictory results obtained from some clinical studies; for example, 229 healthy participants consuming daily 28 g of sea buckthorn berries for 3 months did not show altered levels of serum total, HDL (high-density lipoproteins), LDL (low-density lipoproteins) cholesterol, or changes in the serum triacylglycerol concentrations [11]. In another study, consumption of sea buckthorn berries for 33–35 days showed little effects on plasma cholesterol and triacylglycerol levels in 80 overweight women [12]. 

Characteristic fecal bacterial signature has been found in patients with hypercholesterolemia in comparison to healthy subjects, such as *Anaeroplasma* and *Haemophilus* being negatively correlated to total cholesterol and triglyceride levels and positively to HDL particle size [13]. Sea buckthorn berries abundant in polyphenols are a good source of prebiotic substrate. Due to the low absorption and bioavailability of polyphenols in the small intestine, a significant part of dietary polyphenols reaches the colon and is metabolized by gut microbiota [14]. An in vitro study has shown regulating the effect of sea buckthorn berries on gut microbiota by increasing the abundances of *Bacteroides* and *Prevotella* as well as lactic acid bacteria [15]. Furthermore, protein extracted from sea buckthorn seed has also shown prebiotic properties by increasing the abundances of *Clostridiumcoccoides*, *Bifidobacterium*, and *Lactobacillus* in diabetic mice [16]. 

However, the gut microbiota-regulatory effect of sea buckthorn berries on humans has not been studied. Due to the contradictory and inconclusive results from the interventions of sea buckthorn berries in humans, the plasma metabolomics and sequencing technics capable of revealing changes in small organic molecules and gut microbiota provide different perspectives to investigate the effect of sea buckthorn berries. To the best knowledge of the authors, this is the first study to explore the effects of sea buckthorn berries on plasma metabolomic profile and gut microbiota composition in hypercholesterolemic populations.

## 2. Materials and Methods

### 2.1. Ethics

This study protocol was registered as ChiCTR1800014406 at www.chictr.org.cn (accessed on 16 August 2022) and approved by Peking University Institution Review Board (Ethical approval number: IRB00001052-17052). Written informed consent was obtained from all subjects.

### 2.2. Study Participants and Intervention

A total of 56 participants were recruited in this interventional trial through online advertising and by handing out leaflets in the residential areas nearby Peking University Health Center. The details of inclusion and exclusion criteria were listed in our previous publication [17]. Briefly, males and postmenopausal females aged 50–70 years old with (1) serum total cholesterol between 5.2–7.2 mmol/L; (2) insulin less than 25 mU/L; (3) blood pressure less than 160/99 mmHg; (4) hemoglobin less than 120 g/L; (5) thyroid-stimulating hormone between 0.3–4.2 mU/L; (6) alanine aminotransferase less than 60 U/L; and (7) creatinine was less than 115 mmol/L. The exclusion criteria included the diagnosis of diabetes, thyroid diseases, renal diseases, hematological diseases, hepatic dysfunction or myocardial infarction, as well as use of cardiovascular medication, lipid-lowering drugs or dietary supplements, people with persistent inflammatory disease or mental illness, people who cannot participate in this study independently.

All participants were asked to take a bottle of of sea buckthorn berries (*Hippophae rhamnoides* L. subsp. *Sinensis*) puree (30 g) three times a day for 90 days. They were told to keep the same previous dietary habits and physical activities. However, the participants were advised to avoid taking other dietary supplements during the study. Fasting blood samples were collected by an experienced technician from 8:00 to 10:00 AM at day 0, 45, and 90, and then were centrifuged at 3000× *g* for 15 min and stored at 80 °C until use. 

### 2.3. Biochemical Parameters

Plasma ApoA1 and ApoB were measured by using a Hitachi 7170A/7180 Biochemical Analyzer (Hitachi, Japan). The plasma IL-6 and TNF-a level was determined with an ELISA kit (Nanjing Jiancheng Bioengineering Institute, Nanjing, China). Malondialdehyde (MDA) was measured by a determination kit (Wanleibio, Shenyang, China).

### 2.4. ^1^H NMR Metabolomic Analysis

Proton nuclear magnetic resonance spectroscopy (^1^H NMR) metabolomics analysis was performed from plasma by following the protocol described previously [18]. The samples were prepared and analysed in a randomized order. Briefly, an aliquot of 220 µL plasma was mixed with 440 µL phosphate buffer (90 mmol/L NaH2PO4, pH = 7.4) containing 15% D_2_O. After centrifuging, 600 µL of the resulting supernatants were transferred into 5-mm NMR tubes. The NMR experiments were performed at 298 K on a 600 MHz Bruker Avance-III NMR spectrometer (Bruker BioSpin AG, Fällanden, Switzerland) equipped with a Prodigy TCI cryoprobe and a precooled SampleJet sample changer. Carr–Purcell–Meiboom–Gill (CPMG) pulse sequence was used. Metabolites were identified based on 1D CPMG NMR chemical shifts reported in Chenomx NMR Suite 7.5 software (Chenomx Inc., Edmonton, AB, Canada), 2D NMR (^1^H−^13^C heteronuclear single-quantum correlation spectroscopy (HSQC) and ^1^H−^1^H correlation spectroscopy (COSY)), and the metabolite database Human Metabolome Database (HMDB, http://www.hmdb.ca, accessed on 16 August 2022). The Representative CPMG spectrum of plasma samples is shown in Figure S1. Altogether, 22 metabolites were identified using Chenomx and 2D NMR (Figures S2 and S3), and their chemical shifts and peak multiplicity are summarized in Table S1.

### 2.5. Collection of Fecal Samples and Analysis of Gut Microbiota

Fecal samples were collected at the day 0 and day 90. 24-hour feces was collected and pooled from 48 participants at each time pointsAround 500 mg of fecal samples from each participant were collected by using disposable collectors and tubes. DNA was extracted from fecal samples of subjects, and 16sRNA Illumina Genome Analyzer was performed using MiSeq platform. The genomic DNA extraction from feces, PCR amplification, and sequencing were conducted by the Institute of Microbiology, Chinese Academy of Science, Beijing, China, and followed the previous protocol described [19]. 

### 2.6. Statistical Analysis

The values were defined as the mean ± standard deviation (SD). The differences between three-time points were analyzed by One-way ANOVA and followed by the *post hoc* Bonferroni test when the data is normally distributed and variances were homogeneous or else the Kruskal–Wallis test and the post hoc Tamhane test were applied, The significance of differences was described as * *p* < 0.05, ** *p* < 0.01, *** *p* < 0.001, and **** *p* < 0.0001 between groups. Linear discriminant analysis (LDA) effect size (LEfSe) was used to discriminate differentially abundant bacterial taxa, bacterial taxa with LDA absolute value >2 were considered differentially abundant bacterial.

## 3. Results

### 3.1. Effects of Sea Buckthorn Puree on Lipid Biomarkers, Lipid Peroxidation Product, and Inflammatory Markers

**Figure 1:**
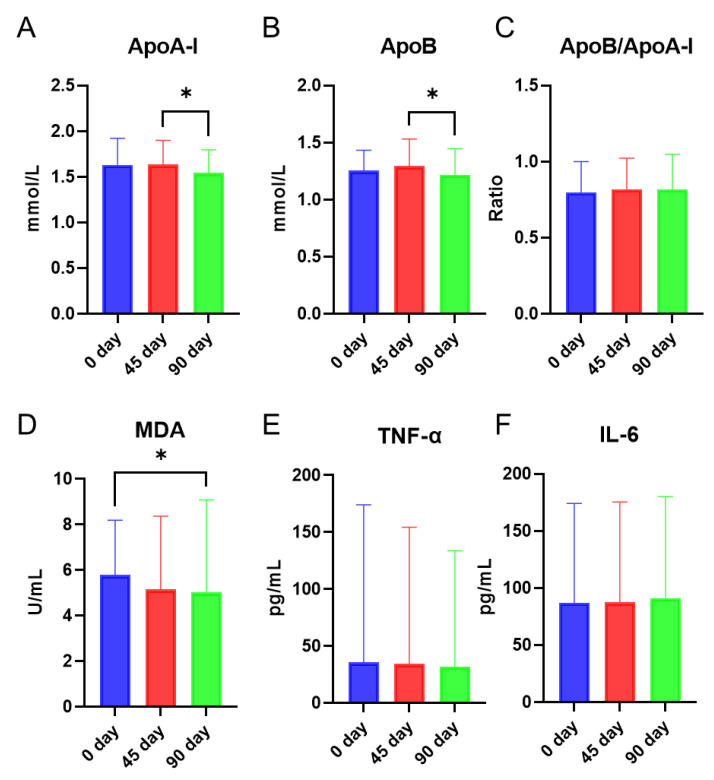
Effects of intervention with sea buckthorn puree on ApoA-I (**A**), ApoB (**B**), Ratio of ApoB/ApoA-I (**C**), malondialdehyde (MDA) (**D**), TNF-α (**E**), and IL-6 (**F**). n = 56, each group. Note: * *p* < 0.05.

In the current study, apolipoprotein (ApoA-I and ApoB), a lipid peroxidation product (malondialdehyde), and inflammatory markers (TNF-α and IL-6) in plasma were studied at baseline and after 45 days and 90 days of intervention. ApoA-I and ApoB remained stable from day 0 to day 45 and significantly decreased at day 90 compared to day 45 (Figure 1A,B). No significant changes were seen in the ratio of ApoB/ApoA-I (Figure 1C). The lipid peroxidation product malondialdehyde (MDA) was significantly decreased at day 90 compared to that at day 0 (Figure 1D). Plasma TNF-α and IL-6 levels were not altered (Figure 1E,F).

### 3.2. Effects of Sea Buckthorn Berries Puree on the Plasma Metabolites 

Twenty-two metabolites including amino acids (branched-chain amino acids, aromatic amino acids, histidine, etc.), compounds related to glucose metabolism (pyruvate, citrate, lactate, etc.), and as well as other metabolites (creatine, creatinine, and acetate, etc.) were identified from plasma ^1^H NMR spectra. 

**Figure 2:**
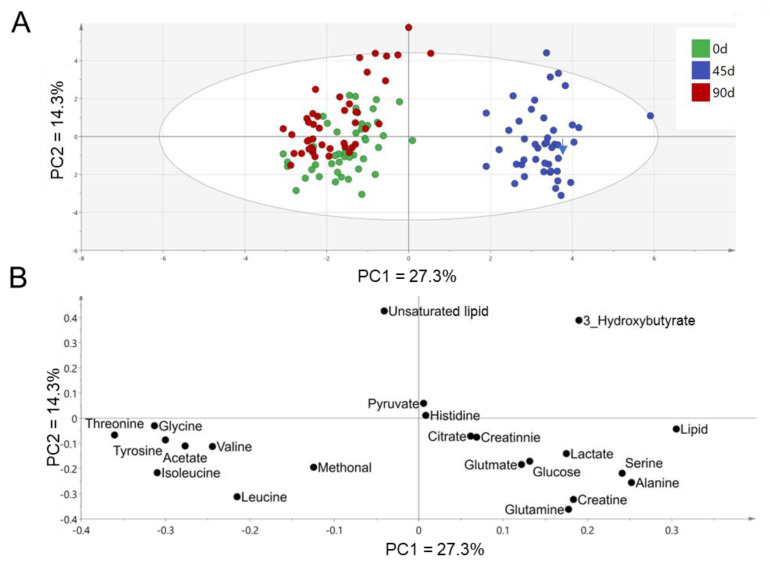
PCA score plot (**A**) and loading plot (**B**) based on metabolites identified from plasma ^1^NMR spectra. n = 44, each group.

Principal component analysis (PCA) was performed based on the plasma metabolic profile to investigate the metabolite alterations associated with the sea buckthorn puree intervention. PCA score plot (Figure 2A) showed a metabolic separation between day 45 and day 0/90 by the first principal component (27.3%), contributed mainly by the altered levels of lipids (resonance generated from CH_3_ protons of fatty acyl chain or fatty acid chain), serine, alanine, threonine, glycine, tyrosine, acetate, leucine, and isoleucine shown in the PCA loading plot (Figure 2B). A slight separation of plasma metabolic profile was found between day 0 and day 90 by the second principal component (14.3%) (Figure 2A), mainly contributed by unsaturated lipid (resonance generated from CH protons of unsaturated fatty acyl chain or fatty acid chain) and 3-hydroxybutyrate (Figure 2B). Overall, plasma metabolic profile was significantly changed from day 0 to day 45, and returned to similar levels as the baseline when the intervention proceeded to day 90.

**Figure 3:**
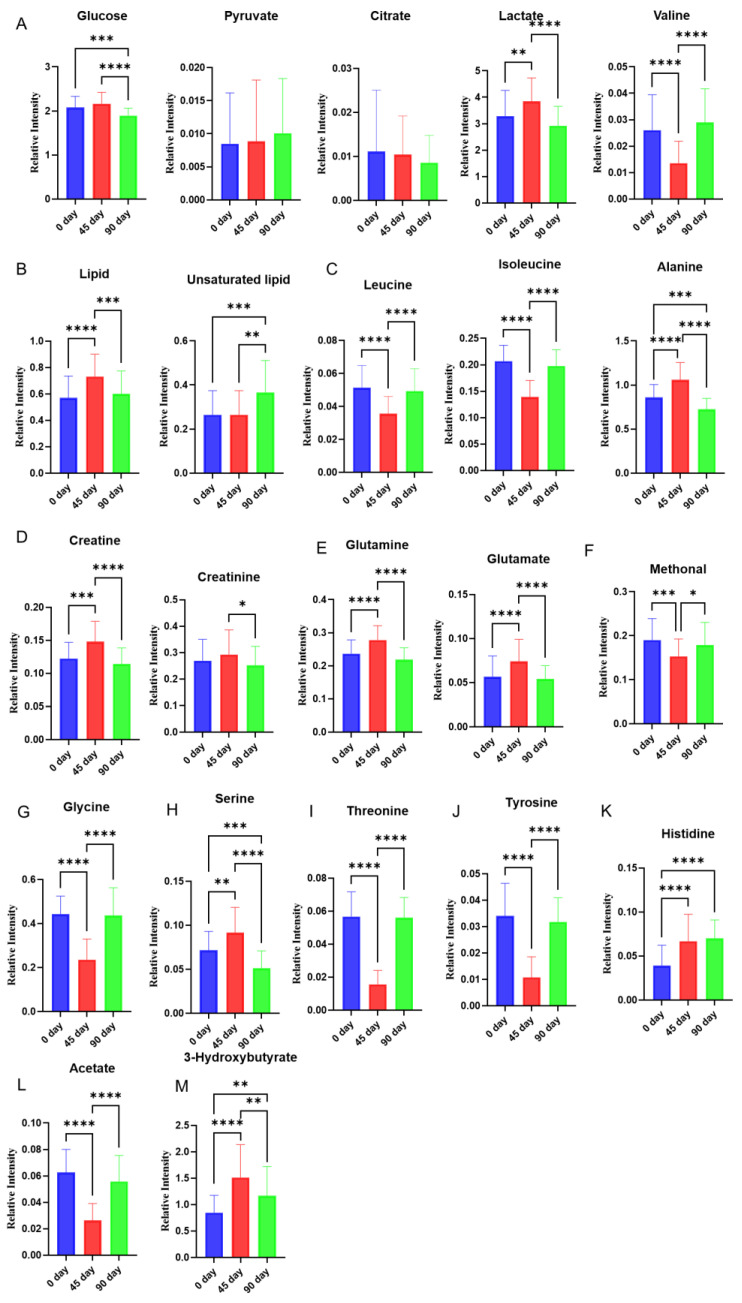
Effect of sea buckthorn puree on plasma metabolites in hypercholesterolemia population. Metabolites involved in glycolysis and TCA cycle (glucose, lactate, alanine, citrate, and pyruvate) (**A**), Lipids (**B**), Branched-chain amino acids (leucine, isoleucine, and valine) (**C**), Creatine and creatinine (**D**), Glutamine and glutamate (**E**), Methanol (**F**), Glycine (**G**), Serine (**H**), Threonine (**I**), tyrosine (**J**), Histidine (**K**), Acetate (**L**), 3-hydroxybutyrate (**M**). n = 44, each group. Note: * *p* < 0.05, ** *p* < 0.01, *** *p* < 0.001, and **** *p* < 0.0001.

After multivariate analysis, histograms are made to show changes in each metabolite at three different time points. Changes of metabolites related to glycolysis and tricarboxylic acid (TCA) cycle were shown in Figure 3A. Glucose level was decreased after 90 days of intervention compared to the level at day 0/45, a similar change was also observed in lactate levels. Valine was decreased at day 45 and then increased at day 90. The lipid level was slightly increased from day 0 to day 45 and then dropped to near the baseline at day 90. Unsaturated lipid level was increased from day 0/45 to day 90 (Figure 3B). 

Branched-chain amino acids (leucine, isoleucine, and valine) were also altered during the intervention. Isoleucine and leucine were decreased during the time point from day 0 to day 45 and then increased to the baseline level at day 90. Alanine was increased at 45 compared to the baseline and then decreased at day 90 to a lower than the baseline (Figure 3C). 

The levels of creatine, creatinine, glutamate, glutamine, serine, and 3-hydroxybutyrate shared similar trajectories, those metabolites were firstly increased at day 45 compared to day 0, and then decreased at day 90 compared to the level at day 45. The levels of creatine, glutamine, serine, and 3-hydroxybutyrate at day 90 were even lower than the baseline (Figure 3D,E,H,M). Methonal, glycine, threonine, tyrosine, and acetate have reversed trajectories compared to the metabolites mentioned above, they were decreased from the baseline to day 45 and then increased back to the level close to the baseline (Figure 3F,G,I,J,L).

### 3.3. Effects of Sea Buckthorn Puree on Gut Microbiota 

**Figure 4:**
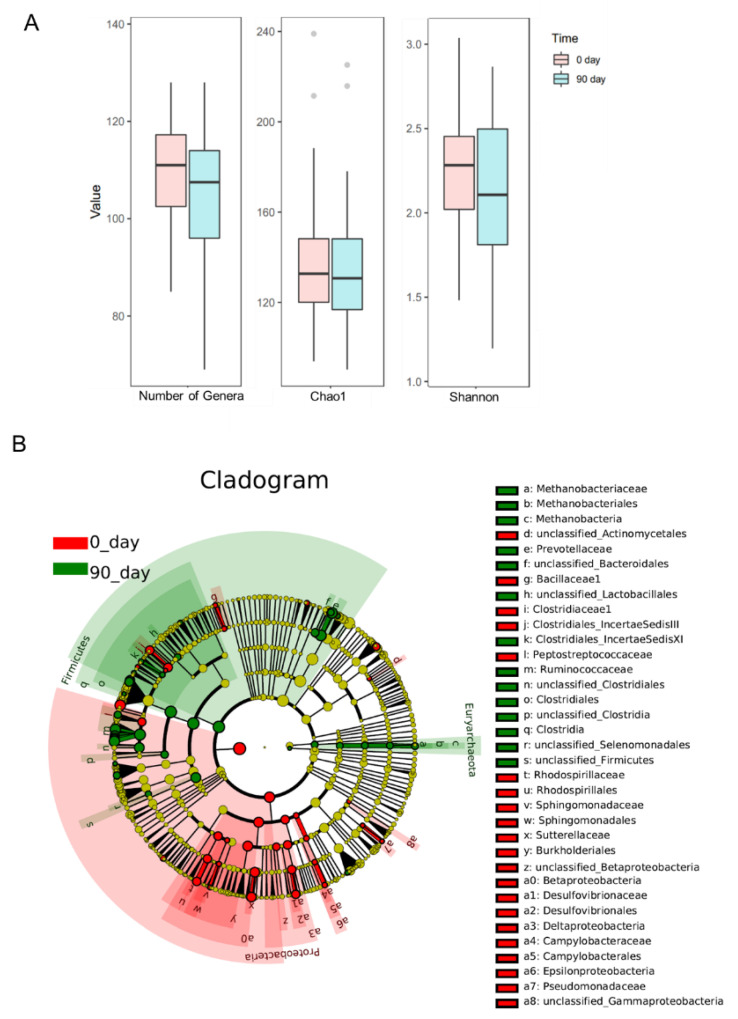
The richness (number of genera) and diversity (Chao1 and Shannon indexes) of gut microbiota (**A**). The structure of the microbiota is presented in the cladograms (**B**), the circle radiated inside-out represents the classification of taxa level from phylum to family. Each small circle at different classification levels represents a taxon and the diameter of the small circle is proportional to the relative abundance. The taxa not with significant differences were colored by yellow and significantly different taxa were colored by different groups, the taxa marked with red color were enriched in the baseline. n = 48, each group.

**Figure 5:**
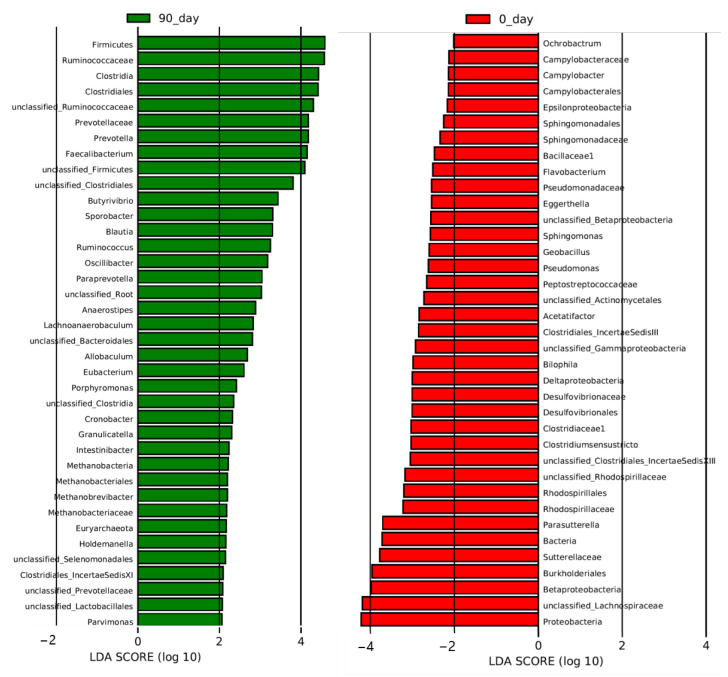
Effect of sea buckthorn berries puree on plasma metabolites in hypercholesterolemia population. From phylum to Genus. The results of LEfSe (LDA Effect Size) analysis level at week 8. The histogram of LDA score generated from LEfSe analysis showed the differentially abundant species (the absolute value of LDA greater than 2) after the treatment of sea buckthorn berries puree. The length of the bars (LDA Score) represents the influencing degree of the species. The species with positive or negative LDA score have higher or lower abundance after the treatment. n = 48, each group.

To analyze the altered gut microbiota composition in the hypercholesterolemia population by the intervention of sea buckthorn puree, 16s RNA sequencing was performed on bacteria in feces collected at day 0 and day 90. The richness and diversity of gut microbiota composition were shown in Figure 4A. The richness of gut microbiota measured as the total number of genera and gut microbial diversity represented as Chao1 and Shannon indexes were not changed after 90 days of intervention of sea buckthorn puree. Cladogram and histogram of LDA score (Figure 4B and Figure 5) generated from LEfSe (LDA Effect Size) analysis showed the different abundance of taxa after treating sea buckthorn berries puree for 90 days. 

To determine the most altered taxa, the histogram of LDA score showed that the top five most upregulated gut microbiota taxa were phylum Firmicutes, family *Ruminococcaceae*, class Clostridia, order Clostridiales, and a genus from the family *Ruminococcaceae* (*unclassified_Ruminococcaceae*) (Figure 5). Phylum Proteobacteria, a genus from the family *Lachnospiraceae* (*unclassified_Lachnospiraceae*), class Betaproteobacteria, class Burkholderiales, and family *Sutterellaceae* were the top five most downregulated gut microbiota taxa by the intervention (Figure 5).

Disbiome database was firstly published in 2018 to provide associations between microorganisms and diseases classified in the Medical Dictionary for Regulatory Activities system. Table 1 summarizes the characteristics of those enriched or depleted genera affected by the intake of sea buckthorn puree [20]. We found that 9 out of 21 enriched genera were butyrate producers which all belonged to the class Clostridiales (*Anaerostipes*, *Ruminococcus*, *Oscillibacter*, *Butyrivibrio*, *Blautia*, *Eubacterium*, *Faecalibacterium*, *unclassified_Ruminococcaceae*, and *Sporobacter*). Only three genera characterized as butyrate producers were depleted after the intervention (*Clostridiumsensustricto*, *unclassified_Lachnospiraceae*, and *Unclassified_Clostridiales_IncertaeSedis XI*). In addition, *Prevotella* and another genus from *Prevotellaceae* (*unclassified_Prevotellaceae*), both of which were high-fiber diet-related probiotics, were also enriched by the treatment. Although two potential pathogens related to metabolic diseases (*Holdemanella* and *Allobaculum*) were enriched by the intervention [21], several potential pathogens were depleted after the treatment, such as *Parasutterella* related to the development of kidney stone and hypertension [22], *Acetatifactor* related to the pathogenesis of obesity and lipid acid metabolism [23], *Eggerthella* associated with coronary heart disease and chronic kidney disease [24], and *Bilophila* related to obesity [25]. 

## 4. Discussion

This is the first study investigating the impact of sea buckthorn puree on plasma metabolic profile and the composition of gut microbiota in the hypercholesterolemic population. Differences in plasma metabolic profiles and gut microbiota were observed before and after the treatment.

Previously we reported the baseline demographic and clinical characteristics of the patients [17]; the total cholesterol was slightly increased from day 0 to day 45 and then decreased to the level close to that of the baseline at the end of the intervention. Soluble vascular cell adhesion molecule-1 was decreased at day 45 compared to level at the baseline and then increased at day 90 [17]. There were no changes in triglycerides, systolic blood pressure, diastolic blood pressure, HDL, LDL, BMI, or high-sensitivity C-reactive protein by the intervention throughout 90 days [17].

ApoB is abundant in very low-density lipoproteins (VLDL) and LDL which are atherogenic particles, leading to the entrapment of these lipoproteins in the arterial wall [26]. ApoA-I is mainly present in HDL particles, which transfer excess cholesterol from peripheral cells to the liver. In addition, ApoA-I exerts anti-inflammatory and antioxidant effects [27]. The ratio of ApoB/ApoA-I has been recognized as a marker for cardiovascular disease, the lower ApoB/ApoA-I ratio is associated with lower risk of cardiovascular diseases [26]. In this study, both ApoB and ApoA-I were decreased from day 0/45 to the end of the intervention, but the ratio of ApoB/ApoA-I was not changed throughout the intervention. Malondialdehyde (MDA) is one of the final products of polyunsaturated fatty acids peroxidation initiated by free radicals [28]. MDA was decreased by the intervention at day 90 compared to the baseline, indicating a possible radical scavenging property of sea buckthorn puree [6]. 

Plasma ^1^H NMR metabolomics revealed that glucose and lactate were increased first from the baseline to day 45, and thereafter their levels dropped all the way till the end of the intervention to the level lower than the baseline. This changing pattern was in line with those of the total cholesterol [17] as well as some detected gluconeogenic amino acids such as alanine, glutamine, and serine found in this study. We speculated that the extra intake of sugar in the sea buckthorn puree and/or increased gluconeogenic amino acids might cause an increase in glucose and its non-oxidative glycolysis product lactate at day 45, nevertheless, the glucose-lowering effect of sea buckthorn puree appeared at the end of the intervention. An increase of lactate is partly contributed by dysregulated pyruvate metabolism, in which pyruvate is converted to lactate rather than acetyl-CoA [29]. Moreover, hypoxia and oxidative stress in skeletal muscle and adipose tissue caused by misfunctioning lactate transporter monocarboxylate transport protein 1 in hypercholesterolemia could also lead to increased lactate [30,31,32]. Decreased glucose and lactate levels at day 90 compared to the baseline might indicate improved glycolysis and oxidative stress by the intervention of sea buckthorn puree, in which polyphenols present in sea buckthorn berries might have played a role [8]. Although the alteration of lipid (resonance generated from CH3 protons of fatty acyl chain or fatty acid chain) was also consistent with the changes in the total cholesterol [17], the unsaturated lipid level (resonance generated from CH protons of unsaturated fatty acyl chain or fatty acid chain) behaved differently and was not altered from the baseline to day 45; however, extension of intervention to 90 days resulted in increase in the level of unsaturated lipids. 

The catabolism of branched-chain amino acids (BCAAs, leucine, isoleucine, and valine) is controlled by a common enzyme branched-chain ketoacid dehydrogenase kinase in skeletal muscle, however, the trajectories of these metabolites changes were not consistent, which might be due to the differential uptake of these amino acids to skeletal muscle [33]. The circulating levels of BCAAs have been reported to be positively correlated with insulin resistance in type 2 diabetes [33]. However, in this clinical study, participants with insulin resistance and type 2 diabetes were excluded. Interestingly, in subjects or animal models without insulin resistance, circulating BCAAs, particularly leucine and isoleucine, might play an important role in regulating glucose metabolism. For example, leucine has been shown to stimulate insulin secretion by deactivating adrenergic α2A receptor via mTOR (Mammalian Target of Rapamycin) pathway in healthy SD rats [34]; Isoleucine has been recognized as a blood glucose-lowering amino acid by increasing glucose uptake in skeletal muscle in healthy Wistar rats [35]. In this study isoleucine and leucine levels were increased from day 45 to day 90, which might partly contribute to lowered plasma glucose from day 45 to day 90 in the population with hypercholesterolemia.

A follow-up cohort study including 4,483 men has shown that men with elevated plasma creatinine level had higher cholesterol and lower HDL levels and were more likely to have cardiovascular disease [36]. In addition, elevated plasma creatinine along with increased plasma cholesterol has been recognized as an indication of kidney malfunction [36]. In this study, the changes of creatinine level were also in line with cholesterol level [17], indicating creatinine can be a potential biomarker for hypercholesterolemia and kidney malfunction. The lowered level of creatinine at day 90 than those at day 0 and day 45 might suggest an improvement of hypercholesterolemia and kidney function by sea buckthorn puree. The relationship between plasma creatinine and hypercholesterolemia deserves further study.

Dietary supplementation of creatine has been reported to be beneficial to lipid metabolism [37]. For example, creatine supplementation (20 g/day for 5 days, followed by 10 g/day for 51 days) decreased the plasma total cholesterol and triacylglycerols without changing plasma LDL and HDL levels huamn [38]. However, both plasma creatine shown in this study and total cholesterol as reported in previous study [17] were found at the highest level at day 45. These results of previous research and the current study indicated that the dietary creatine and endogenous creatine might behave differently in patients with hypercholesterolemia. When the intervention time proceeded to day 90, the plasma creatine decreased to levels below that at the baseline (day 0).

Inhibition of the initiation and progression of atherosclerotic lesions in low-density lipoprotein receptor (LDLR−/−) knock-out mice was associated with the alteration of amino acid metabolism, which was characterized by increased glycine, valine, and glutamine [39]. These results indicate that glycine, valine, and glutamine are involved in the initiation and progression of atherosclerotic lesions. In this study, the levels of glycine and valine decreased at day 45 of the intervention and then increased to the baseline level at the end of the 90-day intervention; however, the glutamine level was first increased and then decreased back to the baseline level. Further research is needed to confirm the possible clinical relevance of such biochemical changes in metabolomic profile. 

Circulating acetate level has been reported to be negatively associated with the levels of blood lipid biomarkers including total cholesterol in 60 hypercholesterolemic adults [40]. Acetate may play a crucial role in brown adipocyte differentiation through the induction of mitochondrial biogenesis, leading to an increased oxygen consumption rate in white and brown adipocytes of mice [41]. An increased oxygen consumption rate was associated with a reduction of reactive oxygen species in hyperlipidemia [42]. Acetate was decreased at day 45 of the intervention period, indicating a possible increase of reactive oxygen species due to the sugar intake [43] via intervention with sea buckthorn puree. As the intervention proceeded to the end of 90-day period, the acetate level fell back to the baseline, suggesting that the functional component in the sea buckthorn berries such as polyphenols possibly decreased reactive oxygen species production [44].

Due to the low absorption, a large proportion of dietary phenolic compounds reaches the colon, where they are metabolized by gut microbiota [14,45]. Polyphenols-gut microbe interactions follow a two-way relationship, with polyphenols shaping the gut microbiota which in turn convert polyphenols into simpler metabolites with increased bioavailability [14,45]. Sea buckthorn berries have been found to increase the abundance of *Bacteroides* and *Prevotella* bacteria in vitro [15]. Intake of freeze-dried sea buckthorn berry powder increased *Akkermansia* and *Bacteroides* in the gut of obese mice [9]. Furthermore, feeding diabetic mice with proteins extracted from sea buckthorn seed increased the abundance of *Clostridiumcoccoides*, *Bifidobacterium*, and *Lactobacillus* [16]. Our present study is the first one to reveal the effect of sea buckthorn puree on the gut microbiota in humans. 

In the present research, we found that intervention with sea buckthorn puree led to alterations in 11 genera of butyrate producers as identified by Disbiome database [20], of which 9 genera were enriched and only 3 genera were depleted by the intervention. Butyrate production in the gut has been reported to be associated with increased gut barrier integrity and improved gut immune responses [46]. Our results showed that supplementation with sea buckthorn puree has the potential to increase the abundance of those butyrate producers (*Anaerostipes*, *Ruminococcus*, *Oscillibacter*, *Butyrivibrio*, *Blautia*, *Eubacterium*, *Faecalibacterium*, *unclassified_Ruminococcaceae*, and *Sporobacter*), which might improve the barrier integrity and immune responses of the gut. 

In this study, the abundance of *Prevotella* and *Faecalibacterium* was increased by sea buckthorn puree in a hypercholesterolemic population. Intake of phenolics has been reported to increase the abundance of *Prevotella* and to enhance the gut barrier integrity in mice [47]. Sea buckthorn berries also increased the abundance of *Prevotella* in vitro [15]. *Ruminococcus* was reported to be associated with glucose and lipid metabolism in postmenopausal women with obesity [48]. In addition, *Prevotella* and *Faecalibacterium* were found to be linked to higher levels of circulating lipids, specifically lysophosphatidylethanolamine and phosphatidylglycerols, which are important intermediates for lipid metabolism and metabolic signaling [49]. These results indicated a possible improvement in the gut barrier as well as glucose and lipid metabolism by sea buckthorn puree in the population with hypercholesterolemia. *Parasutterella* has been reported to be positively associated with BMI, type 2 diabetes, and low-grade inflammation in obesity revealed by a cohort study [50]; however, although *Parasutterella* abundance was deceased by the sea buckthorn puree, the plasma inflammatory markers TNF-α and IL-6 were not altered in population with hypercholesterolemia. High intakes of α-linolenic acid (ALA) were linked to a reduced abundance of *Parasutterella* [50]. Dietary intake of ALA may have beneficial effects on cardiovascular health. The negative association between *Parasutterella* and ALA indicates that regulation of gut microbiota might be a mechanism involved in the positive cardiovascular effects of ALA [51]. The depletion of *Parasutterella* by intervention with sea buckthorn puree indicated the potential benefits to the cardiovascular system in the population with hypercholesterolemia. *Acetatifactor* has been frequently demonstrated to be associated with bile acid metabolism in metabolic diseases [23,52,53]. *Acetatifactor* has been found to be correlated positively with the concentration of secondary bile acids such as lithocholic acid and ursodeoxycholic acid, which can regulate bile acid metabolism and glucose and lipid metabolisms by activating the nuclear farnesoid X receptor and the G protein-coupled membrane receptor 5 [23]. However, in our present study *Acetatifactor* was depleted by the intervention with sea buckthorn puree, of which the mechanism and clinical relevance need more research.

This study revealed the modulatory effects of sea buckthorn puree on plasma metabolomic profile and gut microbiota composition in the hypercholesterolemia population. ^1^H NMR metabolomics was applied to study the plasma samples collected at the baseline as well as after 45 days and 90 days of intervention. The plasma metabolic profile was significantly altered at day 45 compared to the baseline (day 0), as characterized by increased levels of glucose, lipid, lactate, creatine, glutamine, glutamate, serine, and 3-hydroxybutyrate and decreased content of valine, leucine, isoleucine, glycine, threonine, tyrosine, and acetate. As the intervention proceeded to day 90, the general metabolic profile returned to the level similar to the baseline. However, several metabolites remained at different levels from the baseline; for example, the levels of glucose, lactate, creatine, glutamine, and serine remained lower till the end of the 90-day intervention. The extra intake of sugar as part of the sea buckthorn puree could have played a role in influencing energy metabolism in the hypercholesterolemia population leading to an increase in plasma levels of glucose, lactate, and lipids at day 45 of the intervention period, compromising the beneficial effects of sea buckthorn on sugar and lipid metabolism. Nevertheless, the glucose and lactate levels were decreased to the levels lower than the baseline, and lipid levels to the baseline at day 90, which might have been contributed by the action of the bioactive components in the sea buckthorn puree such as phenolic compounds. 

Sea buckthorn puree intervention also modulated gut microbes, such as enrichment of butyrate-producing bacteria and other gut microbes associated with lipid metabolism (*Prevotella* and *Faecalibacterium*) as well as depletion of *Parasutterella* potentially harmful to cardiovascular health. 

Overall, this is the first study investigating the effects of dietary intervention with sea buckthorn puree on plasma metabolomic profile and gut microbiota composition in the hypercholesterolemia population. The findings of the study provide valuable information for further research on the health-promoting effects of sea buckthorn berries. All the participants, whose data were included in the analysis, confirmed that they have consumed the sea buckthorn puree as instructed. However, consumption of sea buckthorn three times a day for 90 days can not be verified for each participant; therefore it can not be excluded that a decreased compliance of the participants during the later phase of the intervention might have caused the revert of the metabolomic profiles towards the baseline. The lack of a control group without intervention is a limitation of this study. Further research using a placebo-controlled study design needs to be conducted to confirm the findings observed in the current study.

## Figures and Tables

**Table 1:** Altered genera affected by sea buckthorn puree and their characteristics generated from Disbiome [20].

Organisms	Characteristics	LDA Score	Regulation
Bacteria. Proteobacteria. Alphaproteobacteria. Rhizobialles. Brucellacese. Ochrobactrum	Oral pathogen	2.01	Downregulation
Bacteria. Firmicutes. Clostridia. Clostridiales. Clostridiaceae1. Clostridiumsensustricto	Butyrate producer	3.03	Downregulation
Bacteria. Proteobacteria. Beta proteobacteria. Burkholderiales. Sutterellaceae. Parasutterella	Potential pathogen, related to kidney stone and hypertension	3.71	Downregulation
Bacteria. Proteobacteria. Gammaproteobacteria Pseudomonadales. Pseudomonadaceae. Pseudomonas	Pathogen, related to many diseases	2.62	Downregulation
Bacteria. Firmicutes. Clostridia. Clostridiales. Lachnospiraceae. unclassified_Lachnospiraceae	Butyrate producer	4.19	Downregulation
Bacteria. Firmicutes. Clostridia Clostridiales. Clostridiales_IncertaeSedisXIll. Unclassified_Clostridiales_IncertaeSedis XI	Butyrate producer	3.05	Downregulation
Bacteria. Firmicutes. Clostridia. Clostridiales. Lachnospiraceae. Acetatifactor	Potential pathogen, related to obesity and lipid acid metabolism	2.83	Downregulation
Bacteria. Bacteroidetes. Flavobacteria. Flavobatteriales. Flavobacteriaceae. Flavobacterium	Pathogen	2.51	Downregulation
Bacteria. Proteobacteria. Alphaproteobacteria. Sphingomonadales. Sphingomonadaceae. Sphingomonas	Potential pathogen, related to many diseases	2.57	Downregulation
Bacteria. Actinobacteria. Actinobacteria. Coriobacteriales. Coriobacteriaceae. Eggerthella	Potential pathogen, related to coronary heart disease and chronic kidney disease	2.54	Downregulation
Bacteria. Proteobacteria. Delta proteobacteria. Desulfovibrionales. Desulfovibrionaceae. Bilophila	Potential pathogen, related to obesity	2.99	Downregulation
Bacteria. Firmicutes. Bacilli. Bacillales. Bacillaceas1. Geobacillus	Potential pathogen, related to psoriasis	2.6	Downregulation
Bacteria. Proteobacteria. Epsilonproteobacteria. Campylobacterales. Campylobacteraceae. Campylobacter	Pathogen	2.13	Downregulation
Bacteria. Proteobacteria. Alphaproteobacteria. Rhodospirilales. Rhodospirillaceae. unclassified_Rhodospirillaceae	Potential pathogen, related to diabetes and celiac disease	3.17	Downregulation
Bacteria. Firmicutes. Clostridia. Clostridiales. Lachnospiraceae. Anaerostipes	Butyrate producer	2.88	Upregulation
Bacteria. Firmicutes. Clostridia. Clostridiales. Lachnospiraceae. Lachnoanaerobaculum	Oral pathogen	2.83	Upregulation
Bacteria. Firmicutes. Clostridia. Clostridiales. Ruminococcaceae. Ruminococcus	Butyrate producer	3.25	Upregulation
Bacteria. Firmicutes. Erysipelotrichia. Erysipelotrichales. Erysipelotrichaceae. Holdemanella	Potential pathogen, related to metabolic diseases	2.15	Upregulation
Bacteria. Firmicutes. Clostridia. Clostridiales. Peptostreptococcaceae. Intestinibacter	Potential pathogen	2.23	Upregulation
Bacteria. Firmicutes. Clostridia. Clostridiales. Ruminococcaceae. Oscilllibacter	Butyrate producer	3.18	Upregulation
Bacteria. Bacteroidetes. Bacteroidia. Bacteroidales. Prevotellaceae. unclassified_Prevotellaceae	Related to high-fiber diet	2.08	Upregulation
Bacteria. Firmicutes. Bacilli. Lactoacillales. Carnobacteriaceae. Granulicatella	Potential pathogen	2.3	Upregulation
Bacteria. Firmicutes. Clostridia. Clostridiales. Lachnospiraceae. Butyrivibrio	Butyrate producer	3.44	Upregulation
Bacteria. Firmicutes. Clostridia. Clostridiales. Lachnospiraceae. Blautia	Butyrate producer	3.3	Upregulation
Bacteria. Firmicutes. Clostridia. Clostridiales. Eubacteriaceae. Eubacterium	Butyrate producer	2.6	Upregulation
Bacteria. Bacteroidetes. Bacteroidia. Bacteroidales. Porphyromonadaceae. Porphyromonas	Oral pathogen, also butyrate producer	2.41	Upregulation
Bacteria. Firmicutes. Clostridia. Clostridiales. Ruminococcaceae. Faecalibacterium	Butyrate producer	4.15	Upregulation
Bacteria. Firmicutes. Clostridia. Clostridiales. ClostridialesIncertaeSedisXl. Parvimonas	Potential pathogen	2.06	Upregulation
Bacteria. Firmicutes. Clostridia. Clostridiales. Ruminococcaceae. unclassified_Ruminococcaceae	Butyrate producer	4.3	Upregulation
Bacteria. Firmicutes. Erysipelotrichia. Erysipelotrichales. Erysipelotrichaceae. Allobaculum	Potential pathogen, related to metabolic diseases	2.69	Upregulation
Bacteria. Bacteroidetes. Bacteroidia. Bacteroidales. Prevotellaceae. Prevotella	Related to high-fiber diet	4.18	Upregulation
Bacteria. Proteobacteria. Gammaproteobacteria. Enterobacteriales. Enterobacteriaceae. Cronobacter	Potential pathogen	2.32	Upregulation
Bacteria. Firmicutes. Clostridia. Clostridiales. Ruminococcaceae. Sporobacter	Butyrate producer	3.31	Upregulation
Archaea. Euryarchaeota. Methanobacteria. Methanobacteriales. Methanobacteriaceae. Methanobrevibacter	Efficient digestion of polysaccharides, producing methane	2.19	Upregulation
Bacteria. Bacteroidetes. Bacteroidia. Bacteroidales. Prevotellaceae. Paraprevotella	Potential pathogen	3.04	Upregulation

## Data Availability

Not applicable.

## Supplementary Materials

The following supporting information can be downloaded at: https://www.mdpi.com/article/10.3390/foods11162481/s1, Figure S1: Representative CPMG spectrum of plasma samples; Figure S2: 2D COSY spectrum; Figure S3: 2D HSQC spectrum; Table S1: Chemical shifts and peak multiplicity of metabolites.
