# Supplementary material for: Dietary Supplementation with Sea Buckthorn Berry Puree Alters Plasma Metabolomic Profile and Gut Microbiota Composition in Hypercholesterolemia Population
Source: Foods. 2022 Aug 17;11(16):2481. doi: 10.3390/foods11162481 (PMC9407212; doi:10.3390/foods11162481)
Supplement: Supplementary file 1 [file foods-11-02481-s001.zip › foods-1826393-supplementary.pdf]

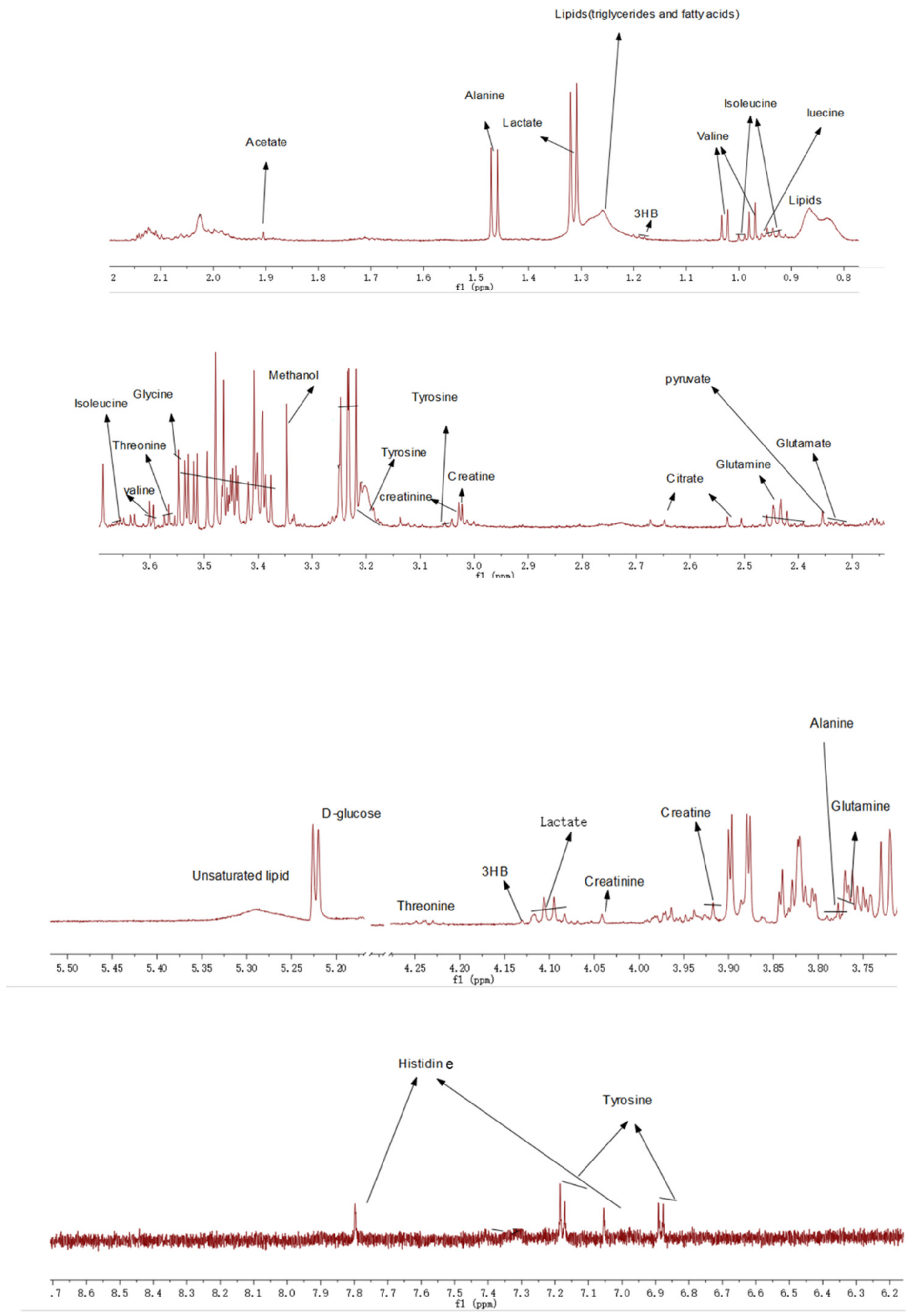

Figure S1 Representative CPMG spectrum of plasma samples

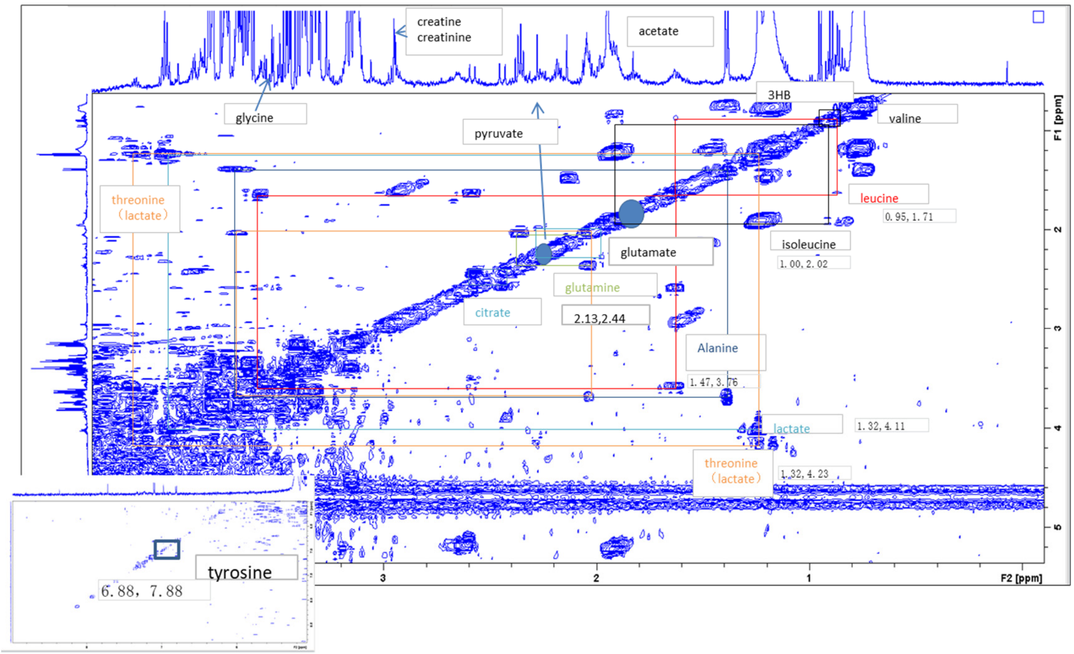

Figure S2 2D COSY spectrum

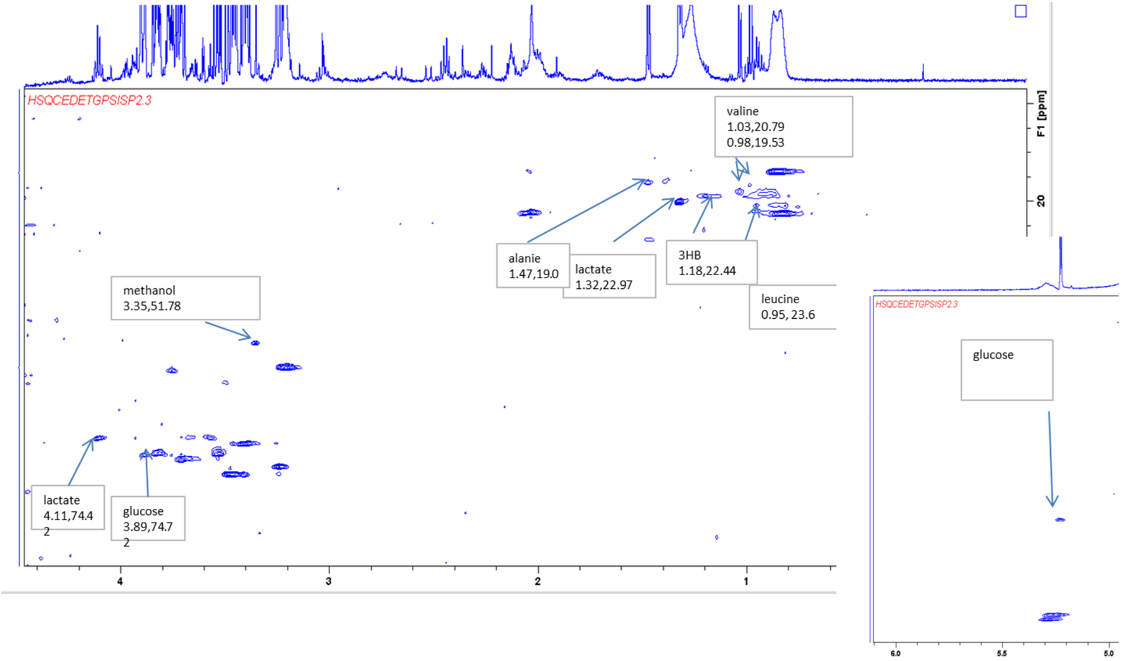

Figure S3 2D HSQC spectrum

Table S1 Chemical shifts and peak multiplicity of metabolites.

No.	Metabolites	δ1H (multiplicity)	δ13C	J value (Hz)	Methods
1	Lipids	0.80-0.90(m)	—	—	CPMG,COSY,HSQC
2	Isoleucine	0.94(t),1.00(d),1.24(m),3.66(dd)	—	—	CPMG,COSY,HSQC
3	Valine	0.98(d).1.03(d),2.26(m),3.60(d)	19.53,20.79	7.01,7.05	CPMG,HSQC
4	Leucine	0.95(t),1.71(m),3.72(dd)	23.6	—	CPMG,COSY,HSQC
5	3-hydroxybutyrate (3-HB)	1.18(d),4.15(m)	22.44	7	CPMG.HSQC
6	Lactate	1.32(d), 4.11(q)	22.97，74.42	7	CPMG,COSY,HSQC
7	Alanine	1.47(d),3.76(q)	19	7.14	CPMG,COSY,HSQC,
8	Acetate	1.91(s)	—	—	CPMG,
9	Glutamine	2.13(m),2.44(m)	—	—	CPMG,COSY,
10	Glutamate	2.05(m),2.34(m)	—	—	CPMG,COSY
11	Serine	3.96(m), 3.83(dd)	—	3.80	
12	Pyruvate	2.36(s)	—	—	CPMG,JRES
13	Citrate	2.52(d),2.66(d)	—	15.5,15.5	CPMG,COSY
14	Creatine	3.02(s)	—	—	CPMG
15	Creatinine	3.03(s)	—	—	CPMG
16	Methanol	3.35(s)	51.78	—	CPMG,HSQC
17	Glycine	3.55(s)	—	—	CPMG
18	Threonine	1.32(s),4.23(s)	—	—	CPMG,COSY
19	Unsaturated lipid	5.3 (b)	—	—	
20	Glucose	3.89(dd),5.226(d)	74.7,95.2	3.88	CPMG,HSQC
21	Tyrosine	6.88(d),7.88(d)	—	8.8	CPMG,COSY
22	Histidine	7.06(s),7.80(s)	—	—	CPMG

s = singlet; d = doublet; dd = double doublet; t = triplet; q = quartet; m = multiplet; br = broad peak
